# B cell receptor signaling in autoimmune rheumatic diseases: regulatory mechanisms and therapeutic targeting

**DOI:** 10.3389/fimmu.2026.1750557

**Published:** 2026-03-04

**Authors:** Yin Zhu, Lu Gao, Yu Han, Fucai Liu, Xin Xie, Xin Dai, Yufen Wang, Yimin Guo, Chunyu Luo, Yan Chen, Pei Huang, Zuochen Du

**Affiliations:** 1 Department of Pediatrics, Affiliated Hospital of Zunyi Medical University, Zunyi, China; 2 Guizhou Children’s Hospital, Zunyi, China; 3 Zhanjiang Institute of Clinical Medicine, Central People’s Hospital of Zhanjiang, Zhanjiang, Guangdong, China

**Keywords:** autoimmune rheumatic diseases, B cell dysregulation, B cell receptor signaling, combination therapies, kinase inhibitors, pathogenesis, precision medicine, therapeutic targeting

## Abstract

Autoimmune rheumatic diseases (ARDs) are a diverse group of chronic disorders characterized by immune dysregulation and multi-organ inflammation. B cell receptor (BCR) signaling emerges as a shared, yet heterogeneously regulated, pathogenic axis across these diseases. This dysregulation drives B cell activation, autoantibody production, and ultimately tissue damage. Recent research highlights its involvement in both common and disease-specific mechanisms, which helps explain the wide variation in clinical features and therapeutic responses across ARDs. This review summarizes current evidence establishing BCR signaling as a central regulatory and therapeutic target in rheumatoid arthritis, systemic lupus erythematosus, Sjögren’s syndrome, IgG4-related disease, and ANCA-associated vasculitis. It integrates mechanistic insights with recent clinical trial data on BCR signaling-targeted therapies, discussing factors that may contribute to variability in therapeutic responses and treatment limitations. Finally, we outline current challenges and future directions for precision medicine in ARDs, with a focus on biomarker-guided strategies and innovative combination therapies to improve patient outcomes.

## 1 Introduction

Autoimmune rheumatic diseases (ARDs) comprise a heterogeneous group of chronic immune-mediated disorders characterized by loss of immune tolerance, autoantibody production, immune complex deposition, and persistent multi-organ inflammation (1). ARDs affect hundreds of millions of individuals worldwide, and their global prevalence, including rheumatoid arthritis (RA), systemic lupus erythematosus (SLE), Sjögren’s syndrome(SS), IgG4-related disease(IgG4-RD), and ANCA-associated vasculitis(AAV), has continued to rise over recent decades, resulting in substantial morbidity, long-term disability, and escalating healthcare burdens (2–10). Despite notable differences in clinical presentation, organ involvement, and disease course, these disorders share a central immunopathological feature: aberrant B cell activation and dysregulated humoral immunity (11).

B cells contribute to ARD pathogenesis through multiple mechanisms, including autoantibody production, antigen presentation, cytokine secretion, and regulation of lymphoid architecture. At the core of these functions lies B cell receptor (BCR) signaling, which governs B cell survival, activation thresholds, differentiation, and tolerance checkpoints (11). Under physiological conditions, appropriate BCR signaling critically regulates B cell fate decisions, thereby maintaining immune homeostasis and enforcing tolerance (12). In contrast, excessive, prolonged, or improperly regulated BCR signaling disrupts central and peripheral tolerance, promotes autoreactive B cell expansion, and drives pathogenic autoantibody responses, ultimately leading to tissue inflammation and damage (13).

Importantly, accumulating evidence indicates that BCR signaling dysregulation represents a shared yet context-dependent pathogenic axis across ARDs. While RA and SLE exemplify classical autoantibody-driven diseases, pSS highlights B cell-mediated glandular destruction and systemic autoimmunity (3). IgG4-RD offers a distinct paradigm of chronic immune activation marked by IgG4-skewed humoral responses, and progressive fibrosis, whereas AAV demonstrates how antigen-specific autoantibodies translate B cell dysregulation into small vessel vasculitis (14). Together, these five conditions span the major clinical and immunological spectra of ARDs in which BCR-dependent mechanisms are both mechanistically implicated and clinically actionable.

From a therapeutic perspective, current ARD management relies largely on broad immunosuppression, including glucocorticoids, conventional disease-modifying antirheumatic drugs, and biologics targeting cytokines or lymphocyte subsets. Although B cell–directed therapies and inhibitors targeting BCR-associated kinases have transformed treatment paradigms in several ARDs, clinical responses remain highly variable (15–19). This heterogeneity underscores fundamental differences in BCR signaling circuitry, disease stage, microenvironmental cues, and interactions with T cells and innate immune pathways (20, 21). Understanding how shared BCR signaling modules diverge across diseases is therefore essential for improving therapeutic precision and overcoming drug resistance.

This review focuses on a cross-disease comparative framework that integrates regulatory mechanisms of BCR signaling with therapeutic translation across multiple ARDs. By systematically comparing RA, SLE, SS, IgG4-RD, and AAV, we aim to delineate both conserved and disease-specific features of BCR signaling dysregulation, critically evaluate emerging and established BCR-targeted therapies, and analyze the mechanistic basis for heterogeneous clinical responses. Furthermore, we highlight current challenges and future directions for precision medicine in ARDs, with particular emphasis on biomarker-guided patient stratification and rational combination strategies. This integrated perspective provides a conceptual framework for understanding BCR signaling as a unifying yet diversified therapeutic axis in autoimmune rheumatic diseases.

## 2 General overview of B cell receptor signaling

### 2.1 Structure of the BCR and signal transduction mechanisms

The BCR is a transmembrane protein complex expressed on B lymphocytes that mediates antigen recognition and initiates adaptive immune responses (22). Structurally, the BCR consists of two major components: a membrane-bound immunoglobulin (mIg) that confers antigen specificity, and an Igα (CD79A)/Igβ (CD79B) heterodimer that is essential for intracellular signal transduction (23, 24). While the variable regions of mIg directly bind antigen, the intracellular domains of Igα and Igβ each contain an immunoreceptor tyrosine-based activation motif (ITAM), which serves as the primary signaling module of the BCR complex (24).

In resting B cells, BCRs are diffusely distributed across the plasma membrane and exhibit limited signaling activity (25). Antigen engagement induces BCR clustering, accompanied by actin cytoskeleton remodeling and the formation of signaling microclusters (26). Beyond classical antigen-induced cross-linking, accumulating evidence indicates that BCR activation can also be driven by antigen-induced conformational changes and mechanical forces generated at the immune synapse, underscoring the mechanistic diversity underlying BCR signal initiation (27). These events trigger the phosphorylation of ITAMs within Igα and Igβ by Src family kinases, most prominently Lyn (22). Phosphorylated ITAMs recruit and activate spleen tyrosine kinase (SYK), which subsequently phosphorylates the adaptor protein BLNK (28). BLNK functions as a scaffold to coordinate downstream signaling by recruiting key effector molecules, including Bruton’s tyrosine kinase (BTK) and phospholipase Cγ2 (PLCγ2) (29). Activated PLCγ2 hydrolyzes phosphatidylinositol 4, 5-bisphosphate (PIP_2_) into two second messengers, inositol trisphosphate (IP_3_) and diacylglycerol (DAG) (30). IP_3_ induces calcium release from the endoplasmic reticulum and promotes sustained calcium influx (31), whereas DAG, together with elevated intracellular calcium, activates protein kinase Cβ (PKCβ) (32). PKC subsequently phosphorylates CARD11, triggering the assembly of the CARD11–BCL10–MALT1 (CBM) signalosome (33). This complex activates IκB kinase (IKK) complex, leading to NF-κB nuclear translocation and transcriptional activation of target genes (34).

In parallel, BCR signaling also regulates cellular metabolism via the PI3K–AKT–mTOR axis (35) and promotes proliferation through the RAS–MAPK pathway (36). Termination and attenuation of BCR signaling are achieved through multiple mechanisms, including receptor internalization, engagement of endocytic adaptors such as LAMTOR2 (37), and the coordinated action of inhibitory phosphatases, ensuring timely signal resolution (38).

In summary, BCR engagement initiates a tightly orchestrated signaling cascade involving ITAM phosphorylation, adaptor protein assembly, and activation of downstream effectors such as BTK, PLCγ2, and NF-κB. Concurrent activation of the PI3K–AKT–mTOR and RAS–MAPK pathways integrate metabolic and proliferative responses. These processes are dynamically regulated to fine-tune signal strength and duration, ultimately determining the magnitude and quality of B cell responses.

### 2.2 Regulatory mechanisms of BCR signaling

The strength and duration of BCR signaling are tightly controlled by an integrated network of positive and negative regulatory mechanisms. Positive regulators amplify BCR signals to ensure effective immune activation. For instance, the CD19–CD21–CD81 coreceptor complex significantly enhances signal transduction efficiency by lowering the activation threshold of B cells (39). Additional positive regulators include DOCK8, which promotes marginal zone B cell differentiation through modulation of CD19 signaling and WASP-dependent cytoskeletal dynamics (40), and STAT3, which facilitates F-actin cytoskeleton accumulation to support BCR microcluster formation (41). Notch2 signaling further augments BCR output by upregulating CD21 expression on the B cell surface (42). Moreover, Fc receptor–like 1 (FcRL1), upon phosphorylation, recruits c-Abl kinase to potentiate BCR signaling at the immune synapse (43).

However, BCR engagement does not always equate to productive activation. In the absence of CD40-mediated (or other) costimulation, BCR triggering can instead drive B cells toward apoptosis rather than activation (44). Thus, CD40 acts as a key checkpoint by providing the additional signal that promotes B-cell survival and enables full BCR-driven activation, helping to set appropriate activation thresholds and tune the strength and duration of downstream signaling (45).

Conversely, negative regulatory mechanisms are essential for restraining excessive B cell activation and maintaining immune tolerance (46). FcγRIIB is a key inhibitory receptor that recruits phosphatases such as SHIP-1 via its ITIM motif, leading to the hydrolysis of PIP_3_ and attenuation of downstream signaling pathways, including those involving SYK and BLNK (47). MAPK4 modulates SHIP-1 expression in an IRF4-dependent manner, providing an additional layer of negative feedback regulation (48). CD22 inhibits BCR signaling by recruiting the SHP-1 phosphatase, thereby limiting calcium flux and dampening B cell activation (49). In addition, cytoskeletal regulators such as ARPC1B contribute to signaling homeostasis by modulating actin polymerization and membrane anchoring, which influence BCR mobility and spatial organization on the cell surface (50, 51).

In summary, BCR signaling is governed by a precisely balanced network of activating and inhibitory regulators that together determine signal strength, duration, and spatial organization. This dynamic equilibrium ensures robust humoral immune responses while preserving self-tolerance. Disruption of these regulatory circuits can lead to abnormal B cell activation and has been increasingly implicated in the pathogenesis of ARDs. A deeper understanding of the molecular mechanisms that fine-tune BCR signaling, will be crucial for the rational development of targeted therapies aiming at restoring immune homeostasis in chronic autoimmune diseases.

## 3 BCR function and dysfunction across autoimmune rheumatic diseases

### 3.1 General mechanisms of BCR dysregulation in ARDs

**Figure 1:**
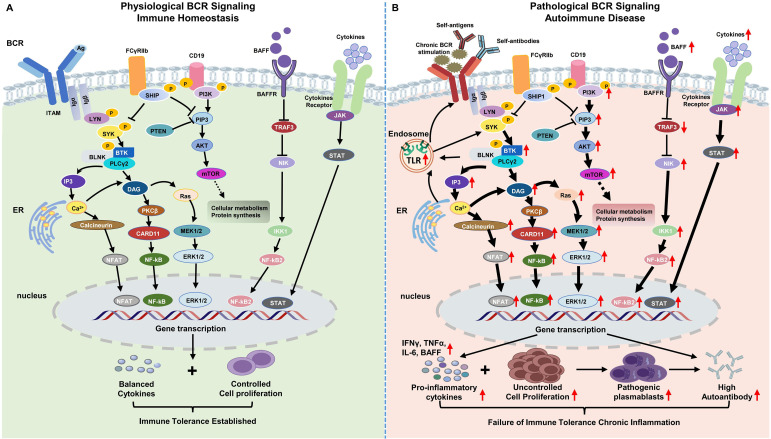
Comparison of Physiological and Pathological B Cell Receptor Signaling in Autoimmune Disease. **(A)** Physiological BCR Signaling in Immune Homeostasis: Antigen binding initiates a tightly regulated cascade involving ITAM phosphorylation by Src family kinases (e.g., Lyn), followed by SYK activation. Downstream signaling through BTK, PLCγ2, and PI3K generates second messengers (IP3, DAG), leading to calcium flux and PKCβ activation. This cascade engages MAPK, NF-κB, and NFAT pathways, promoting controlled B-cell proliferation, differentiation, and protein synthesis. Negative regulators like FcγRIIb and SHIP ensure signal attenuation, maintaining immune tolerance and preventing excessive activation. CD19 co-receptor signaling also modulates the strength of BCR responses. **(B)** Pathological BCR Signaling in Autoimmune Disease: In autoimmune conditions, chronic BCR stimulation, often by autoantibodies and self-antigens, leads to aberrant and sustained activation. This results in prolonged ITAM phosphorylation, enhanced activation of key kinases, and over-activation of downstream pathways (NF-κB, MAPK, PI3K/AKT/mTOR). This pathological signaling drives uncontrolled B-cell proliferation, differentiation into pathogenic plasma cells, and excessive production of pathogenic autoantibodies and pro-inflammatory cytokines. The failure of immune tolerance and chronic inflammation are hallmarks of this dysregulated state. The figure also illustrates synergistic crosstalk between BCR and cytokine receptor signaling, which further amplifies immune activation. The schematic diagram was created using SCI Fig (https://scifig.com).

BCR signaling is a central regulatory pathway governing B cell activation, differentiation, and antibody production under physiological conditions, while also playing a pivotal role in the initiation and maintenance of autoimmune responses (52, 53). In healthy immune systems, tightly regulated BCR signaling contributes to immune homeostasis by ensuring appropriate antigen responsiveness and enforcing tolerance to self-antigens. In contrast, dysregulated BCR signaling under pathological conditions promotes the survival and expansion of autoreactive B cells, excessive autoantibody production, and sustained inflammation, thereby driving the development and progression of ARDs (**Figure 1**).

Aberrant BCR signaling represents a core pathogenic mechanism in ARDs and arises from multiple, interrelated defects in immune regulation (52, 54). One fundamental mechanism is the breakdown of immune tolerance. Defects in central and peripheral tolerance checkpoints allows autoreactive B cells to escape deletion or functional inactivation, enabling their persistence and activation in peripheral tissues (52, 54). In parallel, lowered BCR activation thresholds render B cells hypersensitive to self-antigens, facilitating inappropriate activation and amplification of autoimmune responses even in the absence of strong antigenic stimulation (55, 56). Additionally, prolonged or sustained BCR signaling, often resulting from impaired negative feedback regulation, leads to continuous B cell activation and chronic autoantibody production (56, 57).

BCR dysregulation is further exacerbated by extensive crosstalk with other immune signaling pathways. BCR signaling synergizes with a Toll-like receptor (TLR) pathways (58), as well as downstream effectors such as NF-κB (59), PI3K-AKT-mTOR (60, 61), and JAK–STAT (62) signaling cascades, collectively amplifying immune responses and promoting pathogenic B cell phenotypes. Beyond these intrinsic signaling abnormalities, disease-independent modifiers, including genetic susceptibility, epigenetic alterations, and environmental triggers, can further destabilize BCR signaling networks and accelerate disease progression (63–66).

In addition to these core mechanisms, two interconnected processes critically shape disease outcomes across ARDs. First, immunometabolism reprogramming driven by aberrant BCR signaling, particularly via the PI3K/AKT/mTOR pathway, alters cellular metabolic states to favor B cell survival, proliferation, and sustained autoantibody secretion (67, 68). This metabolic adaptation is especially prominent in diseases such as SLE and RA, where autoreactive B cells persist and drive chronic inflammation (68, 69). Second, the inflammatory tissue microenvironment, characterized by elevated levels of proinflammatory cytokines such as TNF-α and IL-6, further activates autoreactive B cells and accelerates ARD progression (70–72).

In summary, BCR dysregulation in ARDs arises from converging defects in immune tolerance, activation thresholds, signal termination, and pathway integration, collectively sustaining autoreactive B cell survival and chronic autoantibody production. These shared mechanisms establish a pathogenic foundation upon which disease-specific signaling alterations emerge. Understanding these general principles is essential for contextualizing the diverse manifestations of BCR dysfunction observed across individual ARDs and their associated therapeutic implications.

### 3.2 Disease-specific mechanisms of BCR dysregulation

#### 3.2.1 Rheumatoid arthritis

Enhanced BCR signaling activity, characterized by increased phosphorylation of key proximal signaling molecules like SYK and BTK, is a significant pathological feature in RA. This dysregulation, observed in circulating naïve and IgA^+^ memory B cells from autoantibody-positive RA patients (73). It drives the expansion of autoreactive B cell clones, amplifies immune responses, and sustains chronic inflammation within synovial tissues (74, 75).

In parallel, cytokine-mediated signals further potentiate BCR-driven pathogenic processes in RA. IL-6 receptor signaling enhances autoreactive B cells activation, supports the expansion of T follicular helper (Tfh) cells, and contributes to the inflammatory synovial microenvironment (76). The resulting increase in proinflammatory cytokines, including TNF-α and IL-6, exacerbates synovitis, bone erosion, and progressive joint destruction (77, 78).

At the molecular level, activation of the downstream BCR effectors, most notably SYK, BTK, and NF-κB, drives B cell proliferation, differentiation, and inflammatory cytokine production (79, 80). Among these, BTK functions as a central signaling hub, and its sustained activation is closely associated with pathological B cell responses and disease progression in RA (81). Hyperactivation of proximal signaling molecules, including CD19 and BTK (82), leads to persistent engagement of the PI3K–AKT–mTOR pathway (83), disrupting immune homeostasis and promoting excessive cytokine release (74). This sustained signaling also contributes to immunometabolism reprogramming in RA B cells, thereby enhancing their survival expansion, and pathogenic potential (67). Beyond abnormal phosphorylation events, RA B cells often display markedly increased surface IgD levels (84).

Notably, our recent work has identified MAPK4 as an endogenous negative regulator of BCR signaling in RA. MAPK4 expression is significantly reduced in B cells from both RA patients and collagen-induced arthritis models. MAPK4 deficiency enhances aberrant activation of the BCR–PI3K pathway, whereas restoration of MAPK4 suppresses inflammatory B cell populations, limits plasma cell differentiation, and ameliorates arthritis severity. Mechanistically, MAPK4 exerts its inhibitory effects through the IRF4-SHIP-1 axis, highlighting a previously unrecognized regulatory pathway that constrains pathological BCR signaling and disease progression in RA (48).

#### 3.2.2 Systemic lupus erythematosus

Persistent activation of autoreactive B cells, driven by heightened and prolonged BCR signaling, forms a central pathogenic axis in SLE (85). This is reflected by increased phosphorylation of SYK, BTK, and activation of PI3K signaling in B cells from SLE patients, correlating with overall disease activity (86). Functionally, this hyperactivation promotes autoreactive B cell expansion and differentiation into autoantibody secreting cells, thereby reinforcing pathogenic autoantibody responses (87).

Beyond B cell–intrinsic effects, dysregulated BCR signaling amplifies a broader inflammatory network in SLE. Enhanced BCR output strengthens B cell–T cell collaboration (88), augments antigen presentation, and promotes, activation of dendritic cells and macrophages (89), leading to increased production of proinflammatory cytokines, such as IFN-α, IL-6, and TNF-α (90, 91). These interconnected pathways facilitate immune complex formation and deposition in target tissues, ultimately driving multi-organ inflammation and damage (91, 92).

Mechanistically, SLE-associated BCR dysregulation arises from converging defects in tolerance enforcement and inhibitory feedback. Failures in central and peripheral tolerance checkpoints allow the survival and peripheral persistence of highly autoreactive B cell clones (93). In parallel, impairment of key negative regulatory pathways, such as reduced expression or function of FcγRIIB (94) and abnormal activity of SHP-1 (95), weakens feedback inhibition and contributes to dysregulated B cell activation, promoting excessive B cell proliferation, plasma cell differentiation, and sustained autoantibody secretion (92).

The inflammatory microenvironment further lowers the threshold for autoreactive activation. Elevated BAFF levels in SLE provide potent survival and co-stimulatory cues, synergizing with BCR signaling to maintain autoreactive B cell pools and prolong autoantibody production (96, 97). Moreover, epigenetic reprogramming, including altered chromatin accessibility and transcription factor occupancy at BCR-associated loci (98), can prime B cells for hyperresponsiveness (99), thereby stabilizing pathogenic signaling states and perpetuating disease activity.

#### 3.2.3 Primary Sjögren’s syndrome

Consistent with the prominent B cell hyperactivity that characterizes pSS, increased BCR pathway activity is a core pathogenic feature (100, 101). This enhanced activity has been detected in peripheral naïve B cells and salivary gland-infiltrating lymphocytes from pSS patients, evidenced by elevated expression of CD79A/CD79B (101). As a proximal signaling hub, SYK not only transduces BCR signals but can also integrate inputs from Fc receptors, complement receptors, and integrins, thereby broadening and amplifying inflammatory signaling in tissue settings (101, 102). In pSS, sustained SYK activation promotes B cell proliferation and differentiation, augments autoantibody production, and drives inflammatory cytokine release, collectively contributing to immune dysregulation and glandular injury (100, 101, 103).

Importantly, a distinctive feature of pSS is the formation of tertiary lymphoid structures, including germinal center-like reactions, within the salivary glands, which provide a specialized microenvironment supporting persistent BCR engagement, clonal B cell expansion, and overproduction of autoantibodies by autoreactive B cells (103, 104). Within these organized aggregates, B cells undergo antigen-driven clonal proliferation, somatic hypermutation, affinity maturation, and survival signaling, processes reliant on enhanced BCR signaling (105). This tissue-restricted amplification of BCR activity not only drives pathogenic B cell clones but also underpins the chronicity, glandular destruction, and organ specificity of the disease (106).

The BCR–SYK axis is further potentiated by cytokine-dependent threshold modulation and loss of inhibitory constraints (103). Although circulating BAFF levels are frequently increased in pSS, BAFFR expression on B cells is often reduced, suggesting chronic ligand exposure and receptor remodeling in the context of persistent stimulation (107). Functionally, prolonged BAFF signaling lowers B cell activation thresholds and supports the survival of autoreactive clones that would otherwise be restrained by tolerance checkpoints (108). In parallel, dysregulation of CD72-mediated signaling. manifested by an increased proportion of CD72⁺ B cells and elevated serum soluble CD72 (sCD72), may contribute to defective inhibitory control of BCR signaling and thereby promote autoreactivity in pSS (109). Complementing these intrinsic signaling abnormalities, defective IL-10 production by regulatory B cells (Bregs) together with exaggerated T follicular helper (Tfh) responses have been documented in pSS, a disequilibrium that may perpetuate pathogenic B-cell activation and autoantibody production (110).

In addition to adaptive immune amplification, BCR–SYK signaling in pSS intersects with innate immune pathways centered on endosomal Toll-like receptors (TLR7 and TLR9). BCR-driven activation can synergize with TLR7/9-mediated innate immune signaling, and this convergence can further engage downstream programs including JAK–STAT signaling, collectively enhancing B cell activation and inflammatory output (62). Together with inhibitory receptors and checkpoint pathways, these multilayered interactions shape the magnitude and persistence of pathogenic B cell responses in pSS (111).

#### 3.2.4 IgG4-related disease

BCR signaling contributes to the chronic progression of IgG4-RD by promoting antigen-driven B cell activation, clonal expansion, and progressive diversification of antibody responses (112). Aberrant activation of this pathway supports dysregulated B cell proliferation, enhanced IgG4 class switching, and excessive plasma cell differentiation, ultimately resulting in sustained production of pathogenic IgG4 autoantibodies (113). Beyond humoral abnormalities, BCR-driven B cell activation has also been implicated in the development of tissue fibrosis, a defining pathological feature of IgG4-RD (113).

A hallmark of active IgG4-RD is the marked expansion of circulating plasmablasts, which correlates closely with disease activity and extent of organ involvement, rendering this population a robust biomarker for disease monitoring (114, 115). These plasmablasts exhibit heightened protein synthesis and antigen-processing capacity, consistent with persistent upstream BCR engagement and ongoing antigenic stimulation (114, 115).

IgG4 antibodies possess unique structural and functional properties that distinguish them from other IgG subclasses. Notably, IgG4 molecules can undergo Fab-arm exchange, generating bispecific but functionally monovalent antibodies (116). As a result, IgG4 displays reduced ability to crosslink antigens, activate complement, or engage activating Fcγ receptors, features traditionally associated with anti-inflammatory activity (117). However, in the context of IgG4-RD, chronic and excessive IgG4 production reflects sustained B cell activation rather than immune quiescence. Indeed, IgG4 production is often associated with IL-10-producing regulatory B cells, highlighting a paradoxical scenario in which regulatory immune programs coexist with progressive tissue pathology (117).

Importantly, B cells in IgG4-RD contribute directly to tissue remodeling and fibrosis (118), giving rise to the characteristic storiform fibrosis observed histologically (119). T follicular helper (Tfh) cells, activated through antigen-presentation, provide essential signals that promote B cell differentiation into IgG4-producing plasma cells and sustain BCR-dependent activation loops (120). Concurrently, Th1 and Th2 immune responses synergize with BCR signaling to amplify pathogenic effects and promote the formation of tertiary lymphoid structures (TLS) within affected tissues (121). These organized lymphoid niches enhance local B–T cell crosstalk, reinforce persistent BCR engagement, and thereby drive chronic inflammation and fibrosis in IgG4-RD (120, 121).

#### 3.2.5 ANCA-associated vasculitis

Dysregulated BCR signaling contributes to the selective activation and persistence of pathogenic B cell clones in AAV (122, 123). In contrast to autoimmune diseases characterized by broad B cell hyperactivation, BCR dysregulation in AAV is largely focused on antigen-specific responses against myeloperoxidase (MPO) and proteinase 3 (PR3). Engagement of the BCR by these autoantigens promotes clonal B cell expansion (123) and drives the production of pathogenic ANCAs (124), which represent a central initiating event in disease pathogenesis (122). Failures in central and peripheral tolerance checkpoints allow the survival of these autoreactive B cells, enabling sustained autoantibody production (125).

The generation and maintenance of ANCA responses involve distinct B cell subsets. In MPO^+^ AAV patients, CD27^+^IgM^+^ memory B cells are preferentially enriched among MPO-reactive populations, whereas MPO-specific IgG^+^ cells are relatively infrequent, suggesting unconventional differentiation pathways in ANCA-producing B cells (123). Memory B cells and long-lived plasma cells play critical roles in perpetuating ANCA production and sustaining disease activity (126).

Beyond B cell–intrinsic abnormalities, insufficient immune regulation further facilitates pathogenic BCR responses in AAV (127). Imbalances between regulatory T cells and effector memory T cells favor autoreactive B cell activation and promote ANCA production (128). In parallel, defective clearance of apoptotic neutrophils results in prolonged exposure of MPO and PR3 autoantigens to antigen-presenting cells, increasing the likelihood of BCR engagement by autoreactive B cells (125). Neutrophil extracellular traps (NETs) further amplify this process by simultaneously serving as a source of autoantigens and mediators of vascular injury, thereby establishing a self-reinforcing pathogenic loop (129).

Importantly, BCR signaling in AAV is functionally linked to innate inflammatory pathways, particularly complement system, creating a potent feedback loop that amplifies inflammatory responses. Activation of the ANCA-related BCR signaling axis enhances neutrophil responsiveness and exacerbates vascular inflammation and injury (126, 130). Consistent with this, NF-κB-related genes are upregulated in activated memory B cells from AAV patients, and enhanced NF-κB signaling further contributes to immune-mediated vascular injury (17).

#### 3.2.6 Comparative perspectives on disease-specific BCR dysregulation

To delineate the disease-specific roles of BCR signaling in autoimmune rheumatic diseases, **Table 1** provides a comparative overview of key BCR-related features across RA, SLE, pSS, IgG4-RD, and AAV, including dominant signaling abnormalities, cooperative pathways, current therapeutic strategies, and clinical challenges for each disease. This comparative framework underscores both shared pathogenic principles and substantial disease-specific heterogeneity, highlighting the complexity of translating BCR-targeted interventions across distinct ARDs.

**Table 1:** Comparative overview of BCR signaling dysregulation across major autoimmune rheumatic diseases. .

Disease	Dominant BCR-related features	Key cooperative/amplifying pathways	Main BCR-targeted therapeutic approaches	Clinical reality	Key unmet needs
RA	Enhanced signaling in naive & IgA+ memory B cells; increased SYK/BTK phosphorylation	IL-6–STAT3 axis; Tfh–B cell interactions; PI3K–AKT–mTOR signaling (83, 223)	BTK inhibitors; SYK inhibitors; B cell depletion (e.g., anti-CD20) (83)	Biological activity observed, but inconsistent clinical efficacy of BTK/SYK inhibition (131)	Biomarker-guided patient stratification; therapeutic window definition; rational combination strategies (131)
SLE	Sustained BCR activation in autoreactive B cells; tolerance defects; impaired inhibitory signaling (52)	BAFF/APRIL; TLR7/9; type I IFN–JAK/STAT; epigenetic priming (88, 97)	BTK inhibitors; FcRn blockade; co-signaling modulators (224, 225)	BTK inhibition shows heterogeneous outcomes; BCR-independent mechanisms limit durability (131)	Disease endotyping; predictive biomarkers; combination approaches targeting survival and effector pathways
SS	Salivary gland lymphocytic infiltration; autoantibody production; SYK activation (106)	Excessive BAFF with reduced BAFFR feedback; TLR7–MyD88; JAK–STAT; tissue microenvironment (225)	BTK inhibitors; SYK inhibitors; BAFF/APRIL blockade; B cell depletion (226, 227)	Promising early signals, but tissue-resident B cells & heterogeneity complicate outcomes (131)	Improved tissue targeting; patient stratification; long-term efficacy and safety assessment (131)
IgG4-RD	IgG4+ plasma cell infiltration, fibrosis, specific autoantibodies (115)	BCR signaling, Th1/Th2 imbalance, Tfh–B cell crosstalk (121)	B cell depletion (anti-CD19, anti-CD20); exploratory BTK inhibition (228)	B cell depletion highly effective; relapse linked to B cell repopulation (113)	Optimization of maintenance therapy; infection risk management; endotype-based treatment duration (113)
AAV	ANCA-producing B cell clones; MPO/PR3 specificity; memory B/plasma cell persistence (123, 126, 229)	Complement C5a–neutrophil axis; NF-κB–driven inflammation; defective immune regulation (126)	B cell depletion (rituximab); BAFF inhibition; exploratory BTK inhibitors (126)	Rituximab effective for induction/maintenance; pathway inhibitors remain investigational (126)	Better targeting of ANCA-B cell clones; combination therapies with immunomodulatory strategies (222)

RA, rheumatoid arthritis; SLE, systemic lupus erythematosus; pSS, primary Sjögren’s syndrome; IgG4-RD, IgG4-related disease; AAV, ANCA-associated vasculitis; ACPA, anti-citrullinated protein antibody; BCR, B-cell receptor; BAFF, B-cell activating factor; APRIL, a proliferation-inducing ligand; BTK, Bruton’s tyrosine kinase; SYK, spleen tyrosine kinase; PI3K, phosphoinositide 3-kinase; NF-κB, nuclear factor kappa-light-chain-enhancer of activated B cells; JAK/STAT, Janus kinase/signal transducer and activator of transcription; Tfh, T follicular helper cells; MPO, myeloperoxidase; PR3, proteinase 3; FcRn, neonatal Fc receptor.

**Figure 2:**
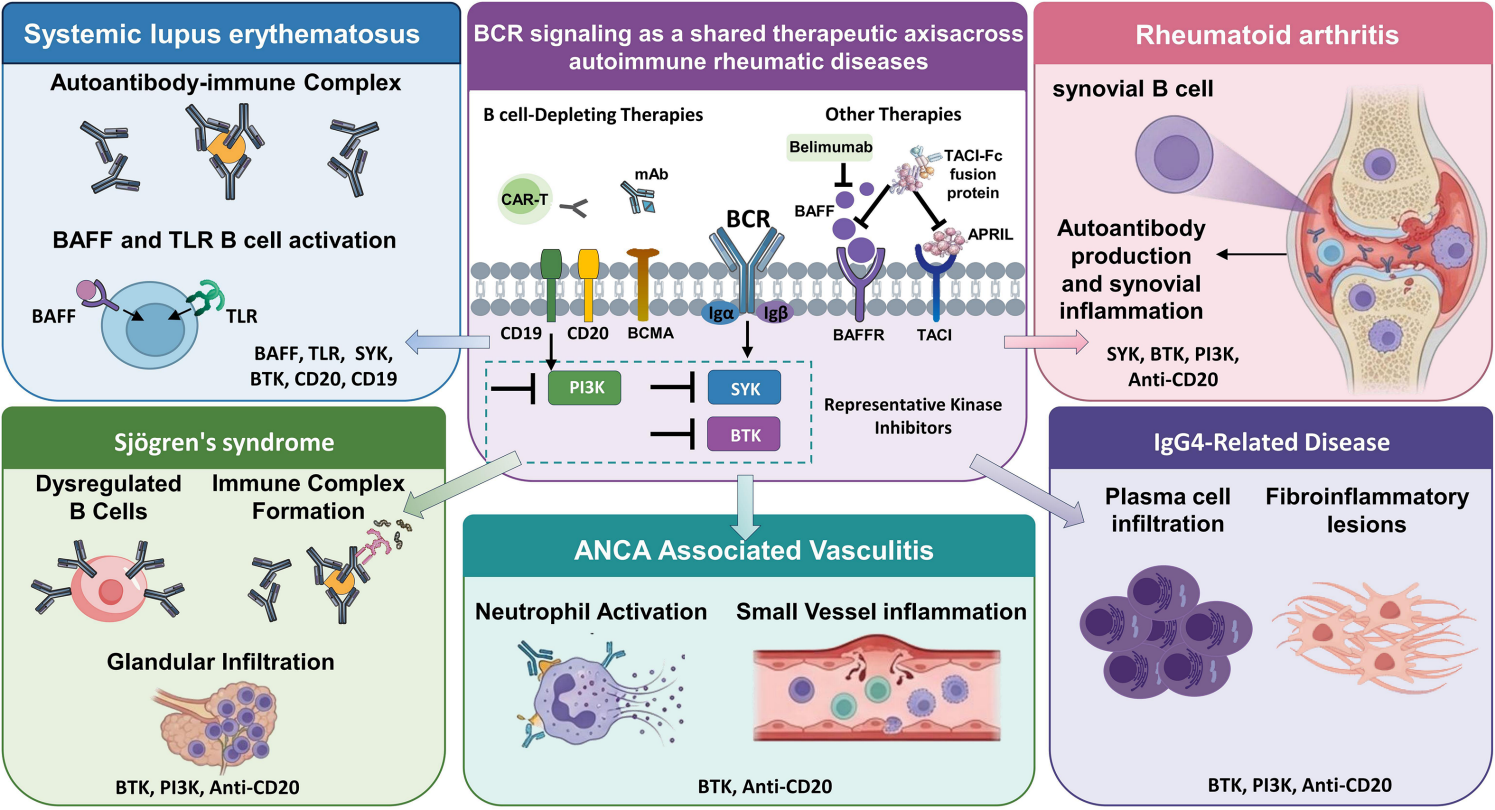
B cell receptor signaling as a shared therapeutic axis across autoimmune rheumatic diseases. This figure illustrates the central role of B Cell Receptor signaling and associated co-receptors (CD19, CD20) as a therapeutic axis in five autoimmune rheumatic diseases: Systemic Lupus Erythematosus, Rheumatoid Arthritis, Sjögren’s Syndrome, IgG4-Related Disease, and ANCA-Associated Vasculitis. Key targets within the BCR pathway, including SYK, BTK, and PI3K, are shown alongside representative therapeutic strategies such as kinase inhibitors and B cell-depleting monoclonal antibodies (e.g., anti-CD19, anti-CD20). For each disease, the figure highlights distinct pathogenic features and the specific BCR-targeted therapies that address these mechanisms. The collective aim of these interventions is to suppress aberrant B cell activation, autoantibody production, and inflammation, thereby advancing precision medicine in ARD management. The schematic diagram was created using SCI Fig (https://scifig.com).

In summary, aberrant BCR signaling emerges as a unifying yet context-dependent pathogenic mechanism in ARDs, underpinning dysregulated B cell activation, autoantibody production, and sustained inflammatory responses. While the core components of the BCR pathway represents attractive therapeutic targets, the manner in which BCR signaling is amplified, integrated with other immune pathways, and constrained by regulatory networks varies markedly among diseases. As illustrated in **Figure 2**, disease-specific BCR-driven pathogenic mechanisms and their corresponding targeted therapies highlight the importance of mechanistic insight in guiding the development of precise and personalized treatments for ARDs.

## 4 Therapeutic targeting of BCR signaling in autoimmune rheumatic diseases

Targeting BCR signaling has emerged as a promising therapeutic strategy for ARDs (131), given the central role of dysregulated BCR activity in the pathogenesis of conditions such as RA, SLE, and pSS, providing a strong mechanistic rationale for therapeutic intervention at this pathway (132, 133). A diverse array of therapeutic agents, including small-molecule inhibitors and biologic therapies, are being developed to modulate B cell activation, autoantibody production, and inflammation (131). But it is important to recognize that many BCR-targeted therapies remain under clinical investigation and have not yet been established as standard first-line treatments for most ARDs (133, 134).

To facilitate a comparative understanding of these strategies, **Table 2** summarizes the major BCR-targeted therapeutic approaches, highlighting their mechanisms of action, clinical limitations, and potential avenues for optimization. Building on this framework, the present section reviews the principal modalities for targeting BCR signaling in ARDs, with a particular focus on proximal and downstream kinase inhibitors, BCR-associated strategies beyond kinase inhibition, and emerging therapeutic approaches currently shaping the translational landscape.

**Table 2:** Targeted therapeutic strategies acting on the BCR signaling axis: mechanisms, challenges, and future directions.

Therapeutic strategy	Target level in BCR network	Representative agents	Clinical development status/key evidence	Best-fit disease contexts	Key limitations	Future optimization directions
BTK Inhibitors	Central amplification node downstream of BCR; links B cell activation to proliferation/survival	Ibrutinib, Orelabrutinib, Fenebrutinib, Evobrutinib, Acalabrutinib, Remibrutinib, Pirtobrutinib	Multiple agents in Ph II/III trials across ARDs; some approved for hematological malignancies (138).	RA (230), SLE (194), pSS (143)	Heterogeneous efficacy in unselected cohorts (131); resistance mutations (e.g., C481S) (140); safety concerns (e.g., bleeding, infection, hepatotoxicity) (131); off-target effects (141).	Non-covalent/reversible inhibitors; PROTAC degraders; biomarker-guided patient selection; combination therapy; improved selectivity (138).
SYK Inhibitors	Proximal BCR signal initiation; integrates FcR signaling	Fostamatinib	Investigational in ARDs; Ph I showed early promise in RA (231), but Ph III failed (165).	RA, pSS	Modest efficacy in later-phase trials (165); narrow therapeutic window; off-target immune suppression; gastrointestinal AEs (165).	Improved selectivity; optimized dosing strategies; combination with downstream modulators or B cell depletion.
PI3Kδ Inhibitors	B cell survival, proliferation, and metabolic adaptation (via AKT/mTOR)	Idelalisib, Leniolisib, Duvelisib	Investigational in ARDs; Leniolisib approved for APDS (161).	SLE, pSS, selected B cell–driven contexts	Infection risk; systemic immunosuppression; tolerability issues; need for patient endotyping.	Intermittent dosing; combinatorial regimens; patient endotyping for optimal selection.
B cell Depletion Therapies	Removal/functional modulation of BCR-expressing B cells (CD19+, CD20+)	Rituximab (anti-CD20), Inebilizumab (anti-CD19)	Rituximab widely used/approved in several ARDs (171, 173); Inebilizumab has Ph III success in IgG4-RD (180).	IgG4-RD, AAV, severe SLE, RA, pSS	Infection risk (221); hypogammaglobulinemia; relapse upon B cell repopulation; limited efficacy in some patient subsets.	Maintenance optimization; monitoring immune reconstitution; targeted depletion of specific B cell subsets; combination with other immunomodulators.
Fc Receptor-Based Therapies	Promotes degradation of pathogenic IgG antibodies by blocking FcRn-mediated recycling	Rozanolixizumab, Efgartigimod, Nipocalimab	Several FcRn inhibitors in Ph II/III for various IgG-mediated diseases (117, 232); some approved (e.g., for gMG) (232).	gMG, pSS, selected IgG-mediated autoimmune diseases (e.g., ITP, PV) (117, 232).	Global reduction in IgG levels (including protective antibodies) (232); potential for increased infection risk (232); long-term safety profile.	Targeted IgG subtype reduction; combination with other B cell pathway inhibitors; patient selection based on autoantibody profiles.
BCR Complex-Directed Agents	Structural disruption of BCR complex and induction of B cell apoptosis	Polatuzumab Vedotin (anti-CD79b ADC)	Approved for hematological malignancies (233); limited direct ARD clinical data.	Primarily B cell malignancies; exploratory in ARDs (185).	Cytotoxicity concerns due to payload (185); systemic exposure in non-malignant B cells; limited autoimmune data.	Careful disease-specific evaluation for ARDs; targeted delivery to autoreactive B cells.
CAR-T Cell Therapy	CD19-targeted T cells for profound B cell depletion and immune reset	Autologous/Allogeneic CD19 CAR-T cells, BCMA-CD19 compound CAR-T	Multiple Ph I/II studies and case series show promising results in refractory ARDs (221, 222).	Refractory SLE (strong evidence) (234), RA (19), pSS, other severe autoimmune diseases (222).	Cytokine release syndrome, neurotoxicity, B cell aplasia (hypogammaglobulinemia), high cost, complex manufacturing, T cell exhaustion, potential for relapse (221, 235).	Allogeneic CAR-T (205), dual-targeted CARs (e.g., CD19/BCMA)(204), secretion of immunomodulators, improved safety profiles, targeted delivery, long-term follow-up studies (206).

ADC, Antibody-Drug Conjugate; AEs, Adverse Events; APDS, Activated PI3K-delta Syndrome; BAFF, B-cell activating factor; BLyS, B-lymphocyte stimulator (alternative name for BAFF); BTK, Bruton’s tyrosine kinase; CAR-T, Chimeric Antigen Receptor T-cell; CRS, Cytokine Release Syndrome; DLBCL, Diffuse Large B-Cell Lymphoma; FcR, Fc Receptor; FcRn, neonatal Fc receptor; gMG, generalized Myasthenia Gravis; ICANS, Immune Effector Cell-Associated Neurotoxicity Syndrome; ITP, Immune Thrombocytopenia; Ph, Phase; PI3K, phosphoinositide 3-kinase; PROTAC, Proteolysis-targeting chimera; PV, Pemphigus Vulgaris; RCT, Randomized Controlled Trial; SLE, Systemic Lupus Erythematosus; SYK, spleen tyrosine kinase; TACI, Transmembrane Activator and CAML Interactor.

### 4.1 BTK inhibitors

BTK is a pivotal kinase within the BCR signaling cascade, directly regulating B cell activation, proliferation and survival (135). Upon BCR engagement, BTK coordinates multiple downstream pathways, including NF-κB, MAPK, and PI3K-AKT signaling, thereby integrating signals that shape B cell fate and effector function (132, 133, 136). Given the central contribution of pathogenic B cells to ARDs, targeting BTK has emerged as a logical and attractive therapeutic target for suppressing aberrant BCR signaling and restraining pathological B cell activation (136, 137), particularly in diseases such as RA and SLE (131, 136).

A range of BTK inhibitors is currently approved for clinical use or under active development. These include first-generation covalent inhibitors, such as Ibrutinib, as well as second-generation agents with improved selectivity and alternative binding modes, including reversible covalent or non-covalent inhibitors such as Rilzabrutinib, Fenebrutinib, Evobrutinib, and Acalabrutinib (131, 136, 138). First-generation covalent inhibitors achieve sustained BTK suppression through irreversible binding to the Cys481 residue but are associated with off-target effects and the emergence of resistance-conferring mutations (139, 140). In contrast, second-generation covalent inhibitors were designed to enhance kinase selectivity, whereas noncovalent BTK inhibitors (e.g., pirtobrutinib) were developed to overcome limitations related to Cys481-dependent resistance, including the C481S mutation (138, 141).

Clinical studies have evaluated the efficacy and safety of BTK inhibitors across multiple B cell–driven autoimmune diseases, with the aim of reducing inflammatory cytokine production, attenuating B cell activation, and improving clinical outcomes (137). For instance, Orelabrutinib, a highly selective irreversible BTK inhibitor, demonstrated promising efficacy in a phase Ib/IIa trial in SLE, achieving SRI-4 response rates of 50-64% at week 12, depending on dosage (142). Similarly, Remibrutinib significantly improved EULAR Sjögren’s Syndrome Disease Activity Index (ESSDAI) scores and showed a trend towards improvement in salivary flow rates in a Phase II randomized controlled trial in pSS (143).

Despite these advances, clinical responses to BTK inhibition have been heterogeneous, particularly in SLE (131). Several phase II trials, including those evaluating Fenebrutinib (144) and Evobrutinib (145), failed to demonstrate consistent clinical benefit in unselected SLE populations, despite strong mechanistic rationale and robust target engagement (131). These outcomes highlight substantial interpatient variability and suggest that compensatory activation of alternative signaling pathways within the BCR network, as well as disease-specific pathogenic mechanisms beyond BCR signaling, may limit therapeutic efficacy (133). Safety concerns have also constrained clinical development; for instance, the BTK inhibitor BMS-986142 was discontinued in RA following a phase II trial due to elevated liver enzymes and lack of superiority over placebo (146).

Resistance and adverse events remain key challenges for BTK-targeted therapy. Acquired mutations in BTK can impair the efficacy of irreversible inhibitors, while alternative pathway activation can undermine long-term disease control (133, 147). In addition, treatment-related toxicities, including bleeding risk and increased susceptibility to infection, narrow the therapeutic window for this drug class (148). To address these limitations, next-generation strategies are being explored, including reversible non-covalent inhibitors, and proteolysis-targeting chimeras (PROTACs) designed to degrade BTK (141, 149). In parallel, preclinical studies have identified naturally derived compounds, such as tricyclic pyranochromenone analogs (150) and bioactive components of traditional herbal medicines (151, 152), that may modulate the BTK–NF-κB axis and ameliorate arthritis in experimental models (83, 150). However, their clinical relevance and safety in human ARDs require extensive further investigation.

In summary, BTK inhibition represents a mechanistically compelling strategy for targeting aberrant BCR signaling in ARDs but is challenged by heterogeneous clinical responses, resistance mechanisms, and adverse effects. Future progress will likely depend on improved patient stratification, deeper understanding of BTK-centered signaling networks across distinct disease contexts, and the rational integration of BTK inhibitors into combination or precision-based therapeutic strategies.

### 4.2 SYK and PI3K inhibitors

SYK and PI3K function as critical downstream and parallel mediators within the BCR signaling network (153, 154), orchestrating B cell activation, proliferation, and survival, and their dysregulation has been implicated in the pathogenesis of multiple autoimmune rheumatic diseases (155, 156). SYK serves as a proximal tyrosine kinase that initiates BCR signal propagation and coordinates multiple inflammatory signaling cascades (157, 158). Whereas the PI3K pathway, acting predominantly through AKT and mTOR, integrates antigen receptor signaling with metabolic reprogramming and immune cell fate decisions (159).

A range of pharmacological inhibitors targeting PI3K and SYK has been evaluated in preclinical models and clinical studies. The PI3Kδ inhibitor idelalisib, originally approved for certain B cell malignancies, exhibits potent immunomodulatory activity (160). Leniolisib, another PI3Kδ inhibitor, has shown encouraging immunoregulatory effects in clinical studies for activated PI3Kδ syndrome (161). While its efficacy is being assessed in ongoing clinical trials for pSS, clinical improvements in pSS symptoms have been mixed, with some trials not demonstrating significant improvements in ESSPRI or ESSDAI after 12 weeks of treatment (162, 163). Evidence also suggests potential synergy when PI3Kδ inhibition is combined with other BCR pathway modulators (164), though specific studies on combination with BTK inhibition in autoimmune diseases require further investigation.

SYK inhibition has likewise attracted interest as a strategy to attenuate aberrant BCR-driven immune responses. Fostamatinib, an oral SYK inhibitor, demonstrated immunomodulatory effects and advanced to phase III evaluation in RA (165). In the OSKIRA-4 trial, fostamatinib achieved higher ACR20 response rates compared with placebo in RA patients not currently taking disease-modifying antirheumatic drugs, although improvements in ACR50/70 responses were modest and accompanied by an increased incidence of adverse events, including hypertension and elevated liver enzymes (166). However, fostamatinib failed to demonstrate sufficient clinical benefit in other phase III studies, leading to discontinuation of fostamatinib development for RA (156, 167). These outcomes highlight the challenges of translating proximal BCR pathway inhibition into durable clinical efficacy.

More broadly, clinical experience with PI3K and SYK inhibitors has underscored several limitations of targeting individual signaling nodes (153, 160). Monotherapy directed against a single pathway often yields only modest clinical therapeutic effects (166). Rapid development of resistance and compensatory activation of parallel signaling circuits within the BCR network and broader immune system remain major obstacles (137). In addition, novel small molecules and naturally derived compounds capable of modulating PI3K, SYK, or related pathways are being explored for their anti-inflammatory and immunoregulatory potential (168). The traditional Chinese medicinal compound sinomenine has demonstrated efficacy in preclinical models of RA by inhibiting the PI3K-Akt signaling pathway (169). It is crucial to note that most of these findings are from *in vitro* or animal studies, and their clinical efficacy and safety in ARD patients require comprehensive human trials.

In summary, PI3K and SYK remain biologically compelling targets for modulating BCR-driven immune responses in ARDs. However, clinical experience to date indicates that effective therapeutic exploitation of these pathways will require improved inhibitor specificity, biomarker-guided patient selection, and rational combination strategies that account for signaling redundancy and disease-specific BCR network architecture.

### 4.3 BCR-associated strategies beyond kinase inhibition

Beyond direct kinase inhibition, an expanding range of therapeutic strategies targeting the BCR axis or its associated pathways has demonstrated promising efficacy in autoimmune diseases (131). These approaches include B cell depletion therapies, antibody–drug conjugates, modulation of co-stimulatory or co-inhibitory molecules, Fc receptor-based therapies interventions, as well as cellular therapies and emerging combination therapies (134).

#### 4.3.1 B cell depletion therapies

B cell depletion represents one of the most established and broadly applied strategies for treating ARDs, directly eliminating autoreactive B cells that drive autoantibody production and immune dysregulation (170). Rituximab, a monoclonal antibody targeting CD20, has been extensively used in RA, SLE, pSS, AAV, and off-label in IgG4-RD (171–176). By depleting CD20^+^ B cells through complement-dependent cytotoxicity and antibody-dependent cellular cytotoxicity (ADCC), rituximab effectively reduces pathogenic B cell populations and modulates downstream immune responses (176). Structural studies have shown that rituximab stabilizes CD20 dimers, enabling clearance through multiple mechanisms (177). Despite its efficacy, prolonged B cell depletion is associated with increased susceptibility to infections and disease relapse following B cell repopulation (178). A retrospective study indicates that rituximab induces long-lasting B cell depletion in AAV, which differs significantly from the repopulation kinetics observed in RA and connective tissue diseases (179), underscoring the need for optimized dosing strategies and improved patient monitoring. Inebilizumab, an anti-CD19 monoclonal antibody, provides broader B cell depletion by targeting earlier developmental stages and plasma cell precursors (180). In the phase III MITIGATE trial, inebilizumab significantly reduced relapse rates in IgG4-RD (180), highlighting its therapeutic potential in B cell–dominant conditions. Beyond CD19 and CD20 targeting, investigational B cell depletion agents addressing other antigens are under evaluation or have faced setbacks (15). Daratumumab, an anti-CD38 monoclonal antibody depleting plasmablasts and long-lived plasma cells, demonstrated substantial clinical responses in two patients with refractory SLE (181) and efficacy in five of six patients with refractory lupus nephritis in a case series (182). In contrast, epratuzumab failed phase III trials (EMBODY 1/2) (183) in SLE despite phase II promise (184), illustrating challenges and ongoing research into alternative B cell lineage targets for ARDs.

#### 4.3.2 BCR complex and Fc receptor-based therapies

Additional strategies aim to disrupt BCR structure or modulate antibody effector pathways. Polatuzumab vedotin, an antibody–drug conjugate targeting CD79b, interferes with BCR integrity and induces B cell apoptosis (185). While primarily developed for lymphoma, its mechanism suggests potential applicability to autoimmune diseases such as SLE (186). Obexelimab, a bispecific antibody targeting CD19 and FcγRIIb, represents an alternative strategy that transiently inhibits B cell activation and may confer therapeutic benefit without the risks associated with prolonged B-cell depletion (184). However, clinical studies in SLE have not demonstrated significant efficacy differences compared with control treatments (187), emphasizing the complexity of translating mechanistic promise into clinical benefit.

#### 4.3.3 Co-stimulatory and co-inhibitory molecule modulation

Therapeutic modulation of B cell co-regulatory receptors offers a strategy to fine-tune BCR signaling rather than eliminate B cells outright. CD22, an inhibitory receptor that dampens BCR signaling, has been explored as a therapeutic target (188). Epratuzumab, a monoclonal antibody targeting CD22, demonstrated immunomodulatory effects and clinical benefit in phase II studies by enhancing inhibitory signaling and reducing B cell activation (189). However, Phase III trials yielded mixed results, highlighting challenges related to patient heterogeneity, endpoint selection, and optimal therapeutic context (183). Co-stimulatory pathways have also been targeted to disrupt T-B cell interactions (190). Abatacept, blocking CD80/CD86-CD28 co-stimulation, is approved for RA and inhibits B cell activation via reduced T cell help (190); SLE trials showed mixed results with serological improvements but limited clinical efficacy (191). CD40-CD40L blockade with dapirolizumab pegol was initially reported to be effective in moderately to severely active SLE but failed to confirm this activity in a subsequent phase II study (192). ICOS-ICOSL inhibition by AMG557 showed safety and potential in early SLE trials, consistent with a mechanism involving the limitation of Tfh-B cell support. (193). These approaches highlight opportunities but underscore needs for biomarker selection and combination strategies to overcome redundancy (176).

#### 4.3.4 Combination therapies

Building on the limitations of single-agent therapy, particularly pathway redundancy, compensatory signaling, and heterogeneous clinical responses, combination strategies are increasingly being explored to achieve deeper and more durable disease control in ARDs (194). Combining PI3K inhibitors, with B cell depletion therapies has been proposed as a strategy for broader immune modulation (195). Within this framework, BTK inhibitors can serve as BCR-pathway anchors that may be paired with depletion-based approaches to target complementary components of B cell–driven immunity (196). Likewise, preclinical data from mouse models of lupus indicate that dual PI3Kδ/γ inhibitor duvelisib inhibitors engage a mechanistically distinct B cell–intrinsic signaling axis that may complement BTK inhibition by targeting parallel nodes to promote immune regulation in SLE and related disorders (164). Beyond depletion-based combinations, regimens integrating BCR pathway inhibitors with additional immunomodulatory nodes, including JAK-STAT or BAFF/BLyS inhibitors, represent promising strategies for more comprehensive and personalized immunomodulation (194, 197). Consistent with this rationale, combination approaches that concurrently restrain BCR signaling and broader inflammatory circuitry, including dual inhibition of BTK and JAK signaling, are under investigation in RA and SLE clinical trials (194). More broadly, these challenges have prompted interest in multi-target strategies, including multi-kinase inhibition targeting PI3K, and SYK, as an approach explored in mouse models of autoimmunity to increase signaling coverage (164, 198).

#### 4.3.5 Other and emerging approaches

Emerging BCR-targeted modalities for autoimmune rheumatic diseases increasingly feature natural products modulating signaling pathways in clinical trials (187, 199). Although a phase III trial did not demonstrate efficacy in maintaining remission in RA (200), preclinical studies suggest that curcumin may modulate NF-κB signaling and suppress STAT1-mediated BAFF expression, a key pathway supporting B cell survival (201). In contrast, for compounds such as Ginsenoside compound K, anti-inflammatory effects and B cell inhibition have been observed primarily in preclinical models, suggesting potential mechanisms that await confirmation in human studies (202). Cellular therapies are also advancing, particularly anti-CD19 CAR-T cells originally developed for B-cell malignancies, which have demonstrated striking efficacy in refractory ARDs like SLE and shown promising potential in RA through deep B-cell depletion (203). Beyond conventional autologous CD19 CAR-T, dual-target strategies such as CD19/BCMA targeting (204). Hypoimmune allogeneic “off-the-shelf” CAR-T products (205) and next-generation “armored” CAR-T designs with immunomodulatory payloads have entered early studies, enhancing accessibility, efficacy, and inflammation control (206).

#### 4.3.6 Summary of BCR-targeted therapeutic strategies

While the preceding sections and **Table 2** summarize the mechanistic basis, clinical development status, and limitations of major BCR-targeted therapeutic strategies, **Table 3** provides a detailed overview of the most recent and impactful clinical trial findings across these diverse therapeutic classes and specific agents. BCR-targeted therapies have significantly advanced autoimmune disease treatment, with initial success from pan-B cell depletion and the subsequent development of specific BTK inhibitors. Second-generation BTK inhibitors show promise in various conditions, and other targets like SYK and PI3K are also evolving. Challenges include drug resistance and off-target effects. Future efforts focus on combination therapies, novel targets, and advanced B cell modulation, such as CAR T cell therapy. Advances in single-cell sequencing and personalized approaches aim for more precise antigen-specific therapies and immune tolerance restoration.

**Table 3:** Latest clinical trial advances in B cell receptor-targeted therapies for autoimmune rheumatic diseases.

Agent	Target/mechanism	Disease (s)	Trial phase/status	Key findings/outcome	Reference(s)
Orelabrutinib	BTK inhibitor (irreversible, highly selective)	SLE	Ph Ib/IIa Completed	Promising SRI-4 rates (50-64%) at Week 12; well-tolerated. Ongoing Ph III development.	Li et al., 2025 (142)
Remibrutinib	BTK inhibitor (selective covalent)	pSS	Ph II RCT Completed	Significantly improved ESSDAI scores and salivary flow rates; generally well-tolerated.	Dörner et al., 2023 (143)
Fenebrutinib	BTK inhibitor (non-covalent)	RA	Ph II Completed	Showed positive data in RA; clinical potential in other ARDs warrants further investigation.	Cohen et al., 2020 (230)
BMS-986142	BTK inhibitor (reversible)	RA	Ph II Completed	Discontinued due to inconsistent efficacy and elevated liver enzymes compared to placebo.	Conaghan et al., 2023 (146)
Fostamatinib	SYK inhibitor	RA	Ph II Completed; Ph III Discontinued	Ph II showed improvements; Ph III did not show significant efficacy over placebo and was discontinued.	Baluom et al., 2012; Genovese et al., 2014 (165, 231)
Inebilizumab	Anti-CD19 mAb (B cell depletion)	IgG4-RD	Ph III Completed	Significantly reduced risk of disease relapse.	Stone et al., 2024 (180)
Nipocalimab	FcRn inhibitor	pSS	Ph II Completed	Improved disease activity scores (e.g., ESSDAI) and reduced IgG/autoantibody levels.	Beydon et al., 2023 (236)
Anti-CD19 CAR-T Cell Therapy	Profound B cell depletion and immune reset	Refractory SLE	Multiple Ph I/II studies & Case series	High rates of drug-free remission (up to 2 years), autoantibody seroconversion, and low toxicity in severely refractory patients.	Mackensen et al., 2022; Mougiakakos et al., 2021; Müller et al., 2024; Wang et al., 2025; Wang et al., 2024 (237–239)
Anti-CD19 CAR-T Cell Therapy (4th Gen)	B cell depletion + local immunomodulation (secreting IL-6/TNFα antibodies)	Refractory RA	Emerging Ph I/II data	Tolerable and efficacious in treatment-resistant RA.	Li et al., 2025 (206)

AAV, ANCA-associated vasculitis; ADC, Antibody-Drug Conjugate; ADCC, Antibody-dependent cellular cytotoxicity; AEs, Adverse Events; APRIL, A proliferation-inducing ligand; BAFF, B-cell activating factor; BTK, Bruton’s tyrosine kinase; CAR-T, Chimeric Antigen Receptor T-cell; CDC, Complement-dependent cytotoxicity; ESSDAI, EULAR Sjögren’s Syndrome Disease Activity Index; FcRn, Neonatal Fc receptor; IgG4-RD, IgG4-related disease; Ph, Phase; PI3Kδ, Phosphoinositide 3-kinase delta; PROTAC, Proteolysis-targeting chimera; pSS, Primary Sjögren’s syndrome; RA, Rheumatoid arthritis; RCT, Randomized Controlled Trial; SLE, Systemic Lupus Erythematosus; SRI-4, Systemic Lupus Erythematosus Responder Index-4; SYK, Spleen tyrosine kinase.

### 4.4 Comprehensive clinical trial landscape

Building upon the disease- and agent-specific clinical trial findings summarized in **Table 3**, a broader view of the current clinical research landscape is essential for contextualizing the development of BCR–targeted therapies in ARDs. To this end, **Supplementary Table S1** provides a comprehensive overview of ongoing clinical trials registered on ClinicalTrials.gov, encompassing a wide spectrum of BCR-directed therapeutic modalities under investigation across RA, SLE, SS, IgG4-RD, and AAV.

As illustrated in **Supplementary Table S1**, the expanding and diverse clinical trial pipeline reflects substantial scientific interest and sustained investment in targeting BCR-dependent immune pathways for ARDs. Notably, the predominance of early-phase trials highlights both the innovation and uncertainty that characterize this rapidly evolving field. In addition to established therapeutic classes, emerging modalities, including CAR-T cell therapies, bispecific antibodies, and next-generation small molecules, are increasingly represented, underscoring a shift toward more precise and mechanism-informed intervention strategies. Collectively, this landscape not only signals the availability of future therapeutic options but also reveals areas of concentrated research activity as well as potential gaps in current treatment paradigms.

In summary, the clinical development of BCR-targeted therapies for ARDs is undergoing rapid expansion, driven by advances in drug design, immune engineering, and translational technologies. The integration of innovative approaches, such as cellular therapies, rational combination regimens, and biomarker-guided strategies, together with emerging analytical tools including single-cell and spatial omics, is progressively enabling more personalized, effective, and durable treatment paradigms. At the same time, the breadth of the ongoing clinical trial landscape emphasizes the need for careful patient stratification, dynamic monitoring of therapeutic responses, and rigorous evaluation of safety profiles. Taken together, these efforts highlight a promising yet complex future for precision targeting of BCR signaling in ARDs, aimed at maximizing clinical benefit while minimizing treatment-associated risks.

## 5 Future directions

The next phase of BCR-targeted therapeutic development will depend on both the refinement of existing strategies and the integration of emerging technologies to enhance precision, durability, and clinical effectiveness (131). Rapid advanced approaches in high-dimensional immune profiling, particularly single-cell and spatial omics technologies, are accelerating the transition from empiric immunosuppression toward mechanism-informed and patient-tailored treatment paradigms in autoimmune diseases (207, 208).

### 5.1 Single-cell and spatial omics

Single-cell RNA sequencing enables high-resolution dissection of immune cell heterogeneity by profiling gene expression at the individual cell level, thereby revealing the diverse and heterogeneous B cell populations present in diseases such as SLE and RA (209, 210). Such analyses facilitate the identification of rare autoreactive subsets, reconstruction of B cell developmental trajectories, identify disease-driving BCR signaling alterations, and help explain variability in therapeutic responses and treatment resistance (211).

Spatial transcriptomics further advances these insights by preserving tissue architecture and local cellular context. By mapping gene expression within inflamed organs, this technology elucidates how B cell-associated signaling networks is shaped by tissue-specific microenvironments and immune cell interactions (212). For example, spatially resolved profiling can identify pathogenic niches within synovial tissue in RA (212, 213) or renal tissue in lupus (214), offering opportunities for localized interventions improved disease stratification.

### 5.2 Biomarker-guided precision therapy

Emerging molecular profiling technologies also provide a powerful platform for biomarkers discovery, enabling more rational patient selection and therapy optimization (207, 215). Molecular profiling can help match patients to the most appropriate B-cell-targeted therapies based on disease-specific alterations and the spatial organization within their B cell compartments (216), and can facilitate dynamic monitoring of disease activity and response to treatment (217), thereby improving long-term disease control.

### 5.3 Combination and novel cellular therapies

Given the redundancy and adaptability of immune signaling networks (218), combination therapeutic strategies are likely to play an increasingly important role in future treatment paradigms (133). BCR signaling, JAK–STAT–mediated cytokine pathways, and co-stimulatory immune checkpoint networks constitute complementary regulatory axes in B cell–driven autoimmunity. Integrating these pathways may represent a rational strategy for broader immunomodulation in autoimmune rheumatic diseases (190, 219). Such combination strategies have the potential to overcome resistance mechanisms and optimize long-term therapeutic outcomes.

Meanwhile, cellular therapies are emerging as potentially transformative approaches for severe or refractory autoimmune disease. CAR-T cell therapies targeting B cell markers such as CD19 are now being explored in autoimmune disease (220). Clinical experiences suggest that CAR-T–mediated depletion of autoreactive B cells may induce sustained remission and address disease relapse associated with B cell repopulation, although long-term safety and optimal patient selection remain to be fully defined (221).

### 5.4 Outstanding challenges and considerations

Despite these advances, several challenges (207, 221) must be addressed to fully realize the promise of next-generation BCR-targeted therapies. Profound clinical and molecular heterogeneity across autoimmune diseases complicates the broad application of precision medicine approaches, as patients with clinical phenotypes may harbor distinct pathogenic drivers (207, 216). Long-term safety remains a critical concern, particularly for strategies involving prolonged B cell depletion, which may increase susceptibility to infection and impair protective immunity. In addition, the cost, technical complexity, and limited accessibility of advanced omics platforms may restrict their widespread adoption, especially in resource-limited healthcare settings.

## 6 Conclusion

Aberrant BCR signaling constitutes a central and multifaceted pathogenic driver across ARDs, orchestrating dysregulated B cell activation, autoantibody production, and sustained inflammation responses. Disruption of tightly regulated signaling networks, including those involving BCR, PI3Kδ, NF-κB, and JAK/STAT pathways, undermines immune tolerance and perpetuates autoreactive immune circuits, ultimately leading to chronic tissue damage and disease progression.

Therapeutic strategies targeting key components of the BCR signaling axis, such as BTK, SYK, and PI3Kδ, have marked a significant advance in the treatment of ARDs, including RA, SLE, pSS. However, clinical experience has revealed substantial heterogeneity in treatment responses, with resistance mechanisms. compensatory signaling, and safety constraints frequently limiting long-term efficacy. These challenges underscore the need to move beyond uniform therapeutic approaches toward more refined, disease- and patient-specific strategies.

Emerging technologies, particularly single-cell transcriptomics and spatial profiling, are poised to transform the precision of BCR-targeted interventions. By enabling high-resolution dissection of B cell heterogeneity and tissue-specific immune microenvironments, these approaches provide critical insights into disease-driving pathways, support biomarker-guided patient stratification, and inform the rational design of combination therapies. Such technological advances are essential for decoding the complex, context-dependent wiring of BCR signaling across diverse ARDs and for predicting individual therapeutic responses.

Looking forward, the successful realization of precision medicine in ARDs will depend on a deeper, mechanistically grounded understanding of BCR signaling regulation and its disease-specific integration with parallel immune pathways. The coordinated application of advanced molecular profiling, targeted therapeutic, and adaptive treatment strategies holds substantial promise for improving clinical outcomes while minimizing treatment-associated risks. Addressing unresolved mechanistic questions, overcoming therapeutic resistance, and developing biomarker- driven, patient-tailored combination regimens will be critical priorities in advancing toward more durable, effective, and personalized management of ARDs.
