# Supplementary material for: B cell receptor signaling in autoimmune rheumatic diseases: regulatory mechanisms and therapeutic targeting
Source: Front Immunol. 2026 Mar 4;17:1750557. doi: 10.3389/fimmu.2026.1750557 (PMC12996067; doi:10.3389/fimmu.2026.1750557)
Supplement: Supplementary file 1 [file Table1.docx]

**Table S1: Overview of ClinicalTrials.gov–Registered Trials for Immune-Modulating Therapies in Autoimmune Rheumatic Diseases**

| **Drug Type** | **Drug Name** | **Target** | **Disease** | **Status** | **NCT Number** |
| --- | --- | --- | --- | --- | --- |
| **Cellular Therapy** |  |  |  |  |  |
| CAR-T Cell Therapy | UCAR T-cell | CD19/BCMA | SLE/AAV | Phase I | NCT07305116 |
| CAR-T Cell Therapy | allogeneic umbilical cord blood-derived CAR-T targeting CD19 and BCMA | CD19/BCMA | SLE | Phase I | NCT07274059 |
| CAR-T Cell Therapy | anti-CD19/BCMA CAR-T cells | CD19/BCMA | RA/SLE/SS/AAV | Phase I | NCT07246096 |
| CAR-T Cell Therapy | RD06-05 CART Cell | CD19/BCMA | SLE/AAV | Phase I | NCT07203404 |
| CAR-T Cell Therapy | Anti-BCMA-CD19 CAR-T cells | CD19/BCMA | IgG4-RD | Phase II | NCT07148791 |
| CAR-T Cell Therapy | CD19-BCMA CAR T cell | CD19/BCMA | SLE/SS | Phase I/II | NCT06947460 |
| CAR-T Cell Therapy | UCAR T-cell | CD19/BCMA | SLE/AAV | Phase I | NCT06941129 |
| CAR-T Cell Therapy | UCAR T-cell | CD19/BCMA | SLE | Phase I | NCT06920433 |
| CAR-T Cell Therapy | BCMA-CD19 CAR-T | CD19/BCMA | SLE/SS/IgG4-RD/AAV | Phase II | NCT06794008 |
| CAR-T Cell Therapy | CD19/BCMA CAR-T Cell | CD19/BCMA | SLE | Phase I | NCT06785519 |
| CAR-T Cell Therapy | RD06-05 CART Cell Injection | CD19/BCMA | SLE/AAV | Phase I | NCT06775912 |
| CAR-T Cell Therapy | BCMA/CD19 CAR-T cells | CD19/BCMA | SLE/SS | Phase I/II | NCT06428188 |
| CAR-T Cell Therapy | CD19/BCMA CAR-T cell therapy | CD19/BCMA | SLE | Phase I | NCT06349343 |
| CAR-T Cell Therapy | CD19-BCMA CAR-T cells | CD19/BCMA | SLE/SS/AAV | Phase I/II | NCT06350110 |
| CAR-T Cell Therapy | anti-CD19 CAR T cell therapy | CD19/BCMA | SLE | Phase I/II | NCT06347718 |
| CAR-T Cell Therapy | CD19 CAR-T cells | CD19 | SLE | Phase I | NCT07233642 |
| CAR-T Cell Therapy | CAR-T Therapy | CD19 | SLE | Phase I | NCT07031713 |
| CAR-T Cell Therapy | CART19 | CD19 | SLE | Phase I/II | NCT06839976 |
| CAR-T Cell Therapy | Anti-CD19 CAR-γδ T | CD19 | SLE/SS/AAV | Phase I/II | NCT06828042 |
| CAR-T Cell Therapy | NKX019 | CD19 | AAV | Phase I/II | NCT06733935 |
| CAR-T Cell Therapy | Metabolically Armed CD19 CAR-T cells | CD19 | SLE | Phase I | NCT06711146 |
| CAR-T Cell Therapy | CD19 CAR-T cells | CD19 | SLE | Phase I | NCT06710717 |
| CAR-T Cell Therapy | CD19 Universal CAR-T cells | CD19 | SLE | Phase I | NCT06691152 |
| CAR-T Cell Therapy | Anti-CD19 CAR T-Cell | CD19 | SLE/AAV | Phase I/II | NCT06685042 |
| CAR-T Cell Therapy | Anti CD19 CAR NK cells | CD19 | SLE | Phase I | NCT06613490 |
| CAR-T Cell Therapy | CD19 CAR-T cells | CD19 | SLE | Phase I/II | NCT06585514 |
| CAR-T Cell Therapy | Rapcabtagene autoleucel | CD19 | SLE | Phase II | NCT06581198 |
| CAR-T Cell Therapy | NKX019 | CD19 | SLE | Phase I/II | NCT06557265 |
| CAR-T Cell Therapy | RD06-04 | CD19 | SLE/AAV | Phase I | NCT06549296 |
| CAR-T Cell Therapy | NKX019 | CD19 | SLE | Phase I | NCT06518668 |
| CAR-T Cell Therapy | IM19 CAR-T cells | CD19 | SLE | - | NCT06508346 |
| CAR-T Cell Therapy | SCRI-CAR19v3 | CD19 | SLE | Phase I | NCT06465147 |
| CAR-T Cell Therapy | anti-CD19 CAR-NK cells | CD19 | SLE | Phase I | NCT06421701 |
| CAR-T Cell Therapy | Obecabtagene autoleucel | CD19 | SLE | Phase I | NCT06333483 |
| CAR-T Cell Therapy | CD19-CAR-DNT cells | CD19 | SLE/AAV | Phase I | NCT06316076 |
| CAR-T Cell Therapy | CNCT19 | CD19 | SLE | Phase I | NCT06316791 |
| CAR-T Cell Therapy | MB-CART19.1 | CD19 | SLE | Phase I/II | NCT06189157 |
| CAR-T Cell Therapy | CAR T-cell therapy | CD19 | SLE | Phase I | NCT06150651 |
| CAR-T Cell Therapy | CABA-201 | CD19 | SLE | Phase I/II | NCT06121297 |
| CAR-T Cell Therapy | CD19 CAR-T cell infusion | CD19 | SLE | Phase I/II | NCT06106906 |
| CAR-T Cell Therapy | CD19 Universal CAR-γδ T Cells | CD19 | SLE | Phase I/II | NCT06106893 |
| CAR-T Cell Therapy | CD19 targeted CAR-T cells | CD19 | SLE/SS/AAV | Phase I | NCT06056921 |
| CAR-T Cell Therapy | anti-CD19 CAR NK cells | CD19 | SLE | Phase I | NCT06010472 |
| CAR-T Cell Therapy | Rapcabtagene Autoleucel | CD19 | AAV | Phase II | NCT06868290 |
| CAR-T Cell Therapy | IMPT-514 CART Cell Injection | CD19/CD20 | SLE/AAV | Phase I | NCT06462144 |
| CAR-T Cell Therapy | CT1190B | CD19/CD20 | SLE | Phase I | NCT06822881 |
| CAR-T Cell Therapy | T Anti-CD70 CAR-T | CD19/CD70 | SLE | Phase I | NCT06946485 |
| CAR-T Cell Therapy | ALLO-329 | CD19/CD70 | SLE | Phase I | NCT07085104 |
| CAR-T Cell Therapy | anti-BCMA/CD70-CAR-T cells | BCMA/CD70 | SLE | Phase I | NCT06934447 |
| CAR-T Cell Therapy | CD20/BCMA-directed CAR-T cells | CD20/BCMA | SLE | Phase I | NCT06249438 |
| CAR-T Cell Therapy | BZE2204 | CD19/CD22/BCMA | RA | Phase I | NCT07174843 |
| **Kinase Inhibitors** |  |  |  |  |  |
| Small molecule inhibition | Rilzabrutinib | BTK | IgG4-RD | Phase III | NCT07190196 |
| Small molecule inhibition | Baricitinib | JAK1/JAK2 | RA | Phase IV | NCT05955066 |
| Small molecule inhibition | Upadacitinib | JAK1 | SLE | Phase III | NCT05843643 |
| Small molecule inhibition | Baricitinib | JAK | RA | Phase III | NCT04870203 |
| Small molecule inhibition | Tofacitinib | JAK | RA | Phase IV | NCT04702256 |
| Small molecule inhibition | Tofacitinib | JAK | SS | Phase II | NCT07281456 |
| **Biologic Agents** |  |  |  |  |  |
| Biological agent therapy | Avacopan | C5aR1 | AAV | Phase IV | NCT06611696 |
| Biological agent therapy | Iptacopan | CFB | AAV | Phase II | NCT06388941 |
| Biological agent therapy | XmAb13676 | CD20/CD3 | RA | Phase I | NCT07230353 |
| Biological agent therapy | Alnuctamab | CD3/BCMA | SLE | Phase I | NCT07219563 |
| Biological agent therapy | CLN-978 | CD3/CD19 | SS | Phase I | NCT07041099 |
| Biological agent therapy | CLN-978 | CD3/CD19 | RA | Phase I | NCT06994143 |
| Biological agent therapy | CLN-978 | CD3/CD19 | SLE | Phase I | NCT06613360 |
| Biological agent therapy | Telitacicept | BAFF/APRIL | AAV | Phase IV | NCT05965284 |
| Biological agent therapy | Telitacicept | BAFF/APRIL | AAV | Phase IV | NCT05962840 |
| Biological agent therapy | Telitacicept | BAFF/APRIL | SLE | Phase I | NCT05687526 |
| Biological agent therapy | Telitacicept | BAFF/APRIL | SLE | Phase II | NCT05680480 |
| Biological agent therapy | Telitacicept | BAFF/APRIL | SLE | Phase IV | NCT05666336 |
| Biological agent therapy | Telitacicept | BAFF/APRIL | SLE | Phase III | NCT05339217 |
| Biological agent therapy | Telitacicept | BAFF/APRIL | SLE | Phase III | NCT06456567 |
| Biological agent therapy | Ianalumab | BAFF-R | SLE | Phase III | NCT06711887 |
| Biological agent therapy | Ianalumab | BAFF-R | SLE | Phase III | NCT06133972 |
| Biological agent therapy | Ianalumab | BAFF-R | SLE | Phase III | NCT05639114 |
| Biological agent therapy | Ianalumab | BAFF-R | SLE | Phase III | NCT05624749 |
| Biological agent therapy | Ianalumab | BAFF-R | SLE | Phase III | NCT05126277 |
| Biological agent therapy | Belimumab | BAFF | SLE | Phase IV | NCT06411249 |
| Biological agent therapy | Belimumab | BAFF | SLE | Phase IV | NCT03543839 |
| Biological agent therapy | Rituximab | CD20 | RA | Phase IV | NCT06906549 |
| Biological agent therapy | Rituximab | CD20 | RA | Phase IV | NCT06003283 |
| Biological agent therapy | Rituximab | CD20 | SLE | Phase IV | NCT05828147 |
| Biological agent therapy | Rituximab | CD20 | AAV | Phase III | NCT03942887 |
| Biological agent therapy | Obinutuzumab | CD20 | SLE | Phase II | NCT05039619 |
| Biological agent therapy | BDB-001 | C5a | AAV | Phase III | NCT07168161 |
| **Combination & Other Therapies** |  |  |  |  |  |
| Other therapy | AB-101 + Rituximab | PD-L1/CD20 | RA/SLE | Phase I | NCT06581562 |
| Other therapy | GSK4527363 | - | SLE | Phase I | NCT06576271 |
| Other therapy | Inebilizumab + Blinatumomab | IgG1κ/CD3/CD19 | RA/SLE | Phase II | NCT06570798 |
| Other therapy | Rituximab + Belimumab | CD20/BAFF | SS | Phase IV | NCT06410833 |
| Other therapy | Abatacept | CD80/CD86/CTLA-4 | RA | Phase III | NCT03414502 |
| Other therapy | Depletion of CD3/CD19 in an autologous stem cell transplant | CD3/CD19 | SLE | Phase II | NCT05029336 |
| Other therapy | CC312 | CD19/CD3/CD28 | SLE | Phase I | NCT07193810 |
| Other therapy | CC312 | CD19/CD3/CD28 | SLE/RA | Phase I | NCT06888960 |
| Other therapy | CC312 | CD19/CD3/CD28 | SLE | Phase I | NCT07177911 |

Abbreviations: RA, rheumatoid arthritis; SLE, systemic lupus erythematosus; SS, Sjögren’s syndrome; IgG4-RD, IgG4-related disease; AAV, ANCA-associated vasculitis; CAR-T, chimeric antigen receptor T-cell therapy; CAR-NK, chimeric antigen receptor natural killer cell therapy; UCAR, universal (allogeneic) CAR; γδ T, gamma delta T cells; DNT, double-negative T cells; CD, cluster of differentiation (e.g., CD19, CD20, CD3, CD22, CD28, CD70, CD80, CD86); BCMA, B-cell maturation antigen; BAFF, B-cell activating factor; APRIL, a proliferation-inducing ligand; BAFF-R, BAFF receptor; BTK, Bruton’s tyrosine kinase; JAK, Janus kinase; C5a, complement component 5a; C5aR1, complement component 5a receptor 1; CFB, complement factor B; PD-L1, programmed death-ligand 1; CTLA-4, cytotoxic T-lymphocyte–associated antigen 4; NCT, ClinicalTrials.gov trial registration number.
